# An engineered immunomodulatory IgG1 Fc suppresses autoimmune inflammation through pathways shared with i.v. immunoglobulin

**DOI:** 10.1172/JCI172980

**Published:** 2024-02-15

**Authors:** Sunny L. Sneed, Brian B. Reese, Ana F.S. Laureano, Sneha Ratnapriya, Isabella Fraschilla, Kate L. Jeffrey, Greg P. Coffey, Pamela B. Conley, Robert M. Anthony

**Affiliations:** 1 Center for Immunology and Inflammatory Diseases, Massachusetts General Hospital, Harvard Medical School, and; 2 Center for the Study of Inflammatory Bowel Disease, Department of Medicine, Massachusetts General Hospital, Harvard Medical School, Boston, Massachusetts, USA.; 3 Nuvig Therapeutics, Redwood City, California, USA.

**Keywords:** Immunology, Autoimmune diseases, Immunoglobulins, Immunotherapy

## Abstract

Immunoglobulin G (IgG) antibodies in the form of high-dose intravenous immunoglobulin (IVIG) exert immunomodulatory activity and are used in this capacity to treat inflammatory and autoimmune diseases. Reductionist approaches have revealed that terminal sialylation of the single asparagine-linked (N-linked) glycan at position 297 of the IgG1 Fc bestows antiinflammatory activity, which can be recapitulated by introduction of an F241A point mutation in the IgG1 Fc (Fc^F241A^). Here, we examined the antiinflammatory activity of CHO-K1 cell–produced Fc^F241A^ in vivo in models of autoimmune inflammation and found it to be independent of sialylation. Intriguingly, sialylation markedly improved the half-life and bioavailability of Fc^F241A^ via impaired interaction with the asialoglycoprotein receptor ASGPR. Further, Fc^F241A^ suppressed inflammation through the same molecular pathways as IVIG and sialylated IgG1 Fc and required the C-type lectin SIGN-R1 in vivo. This contrasted with Fc^Abdeg^ (efgartigimod), an engineered IgG1 Fc with enhanced neonatal Fc receptor (FcRn) binding, which reduced total serum IgG concentrations, independent of SIGN-R1. When coadministered, Fc^F241A^ and Fc^Abdeg^ exhibited combinatorial antiinflammatory activity. Together, these results demonstrated that the antiinflammatory activity of Fc^F241A^ requires SIGN-R1, similarly to that of high-dose IVIG and sialylated IgG1, and can be used in combination with other antiinflammatory therapeutics that rely on divergent pathways, including Fc^Abdeg^.

## Abstract 2

<p>The anti-inflammatory activity of recombinant FcF241A is dependent on DC-SIGN, and its half-life is extended by sialylation.</p>

## Introduction

Immunoglobulin G (IgG) is the most prevalent antibody in mammals and accounts for approximately 75% of the total antibodies in typical human circulation (1). IgGs canonically trigger inflammation following the formation of immune complexes by engaging the major IgG effector pathways, including those mediated by Fc γ receptors (FcγRs) and the classical complement pathway. Paradoxically, IgG can also exhibit antiinflammatory activity and is used in the clinic to treat autoimmune and inflammatory diseases in the form of high-dose intravenous immunoglobulin (IVIG) (2). IVIG is a preparation of IgGs pooled from tens of thousands of donors and is given as an antibody replacement therapy to immunocompromised individuals at low doses (200–600 mg/kg) (3–5). However, when administered at a high dose of 1–2 g/kg, IVIG is immunomodulatory (6–9). Indeed, the high cost of regular infusion combined with recent global shortages of IVIG has underscored the need for an alternative, recombinant therapeutic that mimics the antiinflammatory mechanisms of IVIG.

**Figure 1:**
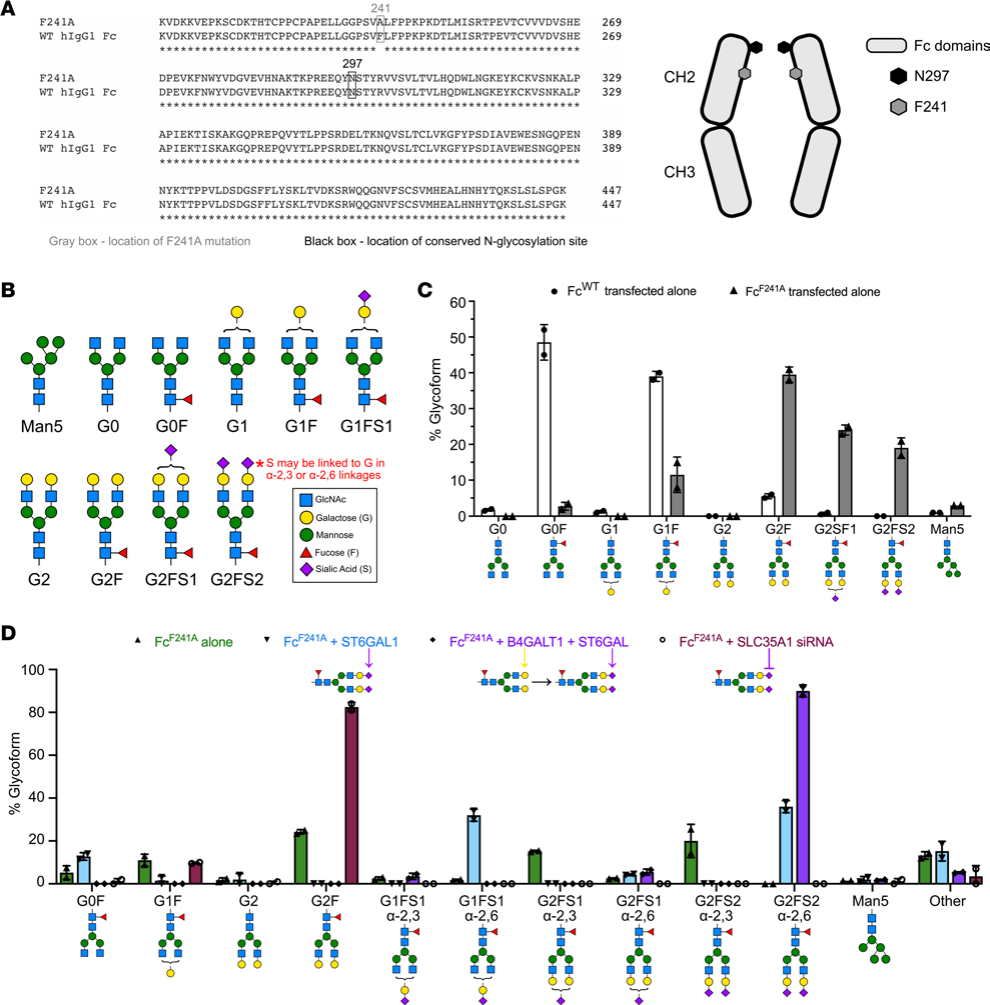
Manipulation of Fc glycosylation of CHO-K1–produced Fc^F241A^. (**A**) Sequence alignment of WT human IgG1 Fc (Fc^WT^) and Fc^F241A^, with the gray box designating residue 241 and the black box designating N297 in both sequences. Graphic of human IgG Fc structure with positions of F241 (gray hexagon) and N297 (black hexagon) marked, showing their relative proximity and location within the interior of the Fc structure. (**B**) Schematic representing the different N-linked glycoforms that may be present on Fc N297 and their nomenclature. Blue squares, *N*-acetylglucosamine (GlcNAc); yellow circles, galactose; green circles, mannose; red triangles, fucose; purple diamonds, sialic acid. (**C**) CHO-K1 cells were transfected with plasmids for either Fc^WT^ or Fc^F241A^, and then N297 glycoforms on the purified Fc products were analyzed via HPLC. (**D**) Percentages of N297 glycans from CHO-K1 cells expressing Fc^F241A^ (green), Fc^F241A^ transfected with ST6GAL1 (Fc^F241A/ST6^, blue), Fc^F241A^ transfected with B4GALT1 and ST6GAL1 (Fc^F241A/B4ST6^, purple), and Fc^F241A^ transfected with siRNA against SLC35A1 (Fc^F241A/siSLC^, maroon). Fc glycoforms were analyzed via HPLC. Bar graphs are plotted as means with SDs in **C** and **D**.

IgG antibodies consist of 2 identical heavy and light chains that form the 2 domains that are crucial for their function (1). The antigen-binding fragment (Fab) is responsible for antigen specificity, while the constant, crystallizable fragment (Fc; Figure 1A) engages effector pathways (10–12) and is responsible for the extended serum half-life by interacting with the neonatal Fc receptor (FcRn) (13–15). All IgGs contain a conserved N297 residue in the Fc portion of the antibody that is posttranslationally modified with a complex-type asparagine-linked (N-linked) glycan, which is necessary for FcγR and C1q binding (16–20). The core glycan structure consists of *N*-acetylglucosamine (GlcNAc) and mannose residues, and may be modified by the addition of fucose, GlcNAc, galactose, and sialic acid (Figure 1B). Indeed, much interest in IgG glycosylation has stemmed from studies that demonstrated the effects of variable glycosylation on Fc function, including afucosylated IgG gaining enhanced binding to FcγRIIIA and more potently triggering antibody-dependent cytotoxicity in vivo than fucosylated IgG (21–25). Additionally, work from our group and others has shown that terminal sialylation of the IgG Fc glycan conveys antiinflammatory activity (26, 27), and notably, approximately 10% of glycans in IVIG are terminally sialylated (1, 26).

The Fc region of IgG is composed of 2 heavy chain domains, CH2 and CH3, that have been shown to adopt either an “open” or a “closed” conformation based on the sugar residues attached to the N-glycan (28–30). It is thought that the glycan stabilizes the local Fc structure, especially in the C′E loop near N297 along a stretch of hydrophobic amino acids (30). Based on these studies, it is proposed that the IgG Fc is typically in an open conformation, which has increased binding affinities for FcγRs, such as FcγRI or FcγRIII. However, when the N297 glycan is terminally sialylated, the Fc adopts a closed conformation, which generally decreases affinities for FcγRs, providing a structural mechanism for antiinflammatory activity (30). Further mechanistic studies have shown that the antiinflammatory activity of sialylated IgG requires DC-SIGN (CD209) or its murine ortholog SIGN-R1 in vivo, which are proposed as antiinflammatory type II FcγRs along with CD23 (1, 31–33).

Removal of terminal sialic acid from IVIG results in loss of protection in animal models of multiple sclerosis, rheumatoid arthritis, Guillain-Barré syndrome, and immune thrombocytopenia (34–36), despite retaining normal circulating half-life and binding to FcRn (26, 37). Conversely, hypersialylation of IVIG increases the potency of antiinflammatory activity 10- to 30-fold in several different animal models of autoimmune disease (38). The biological consequence of binding to and activating the type II Fc receptors is IL-33 release, inhibitory FcγRIIB upregulation, and regulatory T cell expansion (26, 34). In mice, knockout of SIGN-R1 blocks the antiinflammatory activity of IVIG (32). Blocking or deletion of IL-33 receptor or depletion of T regulatory cells (34, 39, 40), in different contexts also blocks the antiinflammatory properties of IVIG.

The phenylalanine residue at position 241 in the Fc has been shown to contribute to thermal and structural stability of the Fc via noncovalent interactions with the N297 glycan (41). Mutation of this residue to non-aromatic amino acids has produced Fcs with increased conformational sampling and N297 glycans with increased mobility and terminal sialylation (30). Importantly, increased mobility and closed conformation sampling of the Fc suggest that the F241A mutation can recapitulate the antiinflammatory properties of sialylated IgG Fc (34, 42). These findings suggest that an IgG1 Fc engineered with F241A mutation (Fc^F241A^) could be developed as an antiinflammatory biological therapeutic.

Another strategy for IgG Fc–based therapeutics is exemplified by the recent development of efgartigimod, a human IgG1 Fc with Abdeg mutations (Fc^Abdeg^) that substantially increase affinity for FcRn (43–45). IgG1 Fc with these mutations has been shown to markedly decrease IgG half-life in mice, cynomolgus monkeys, and humans, especially with repeated dosing, and additional studies have shown its effectiveness in treating myasthenia gravis (43, 46, 47), immune thrombocytopenia (48), and pemphigus (49, 50). Hence, modification of the human IgG1 Fc with F241A or Abdeg mutations appears to confer distinct mechanisms of immune control. Although the FcRn-mediated pathway of action for efgartigimod is well established, other potential background pathways or binding partners are unknown; for example, it has not yet been examined whether efgartigimod is at all reliant on type II Fc receptors such as DC-SIGN.

In the present study, we have characterized the in vivo activities of Fc^F241A^ with varying amounts of sialylation and of Fc^Abdeg^. Our results demonstrate that the antiinflammatory activity of Fc^F241A^ is independent of sialylation level, but in vivo half-life and bioavailability are markedly improved by enhanced sialylation via inhibited interactions with the asialoglycoprotein receptor ASGPR. Fc^Abdeg^ also effectively suppressed autoantibody-mediated inflammation, substantially decreasing endogenous IgG levels as a result of its increased affinity for FcRn relative to Fc^F241A^. IgG suppression was not observed with Fc^F241A^. In addition, we show that Fc^F241A^ and Fc^Abdeg^ inhibit autoantibody-induced inflammation through independent pathways, where IVIG and Fc^F241A^ are dependent on SIGN-R1, while Fc^Abdeg^ is not. Finally, when coadministered, Fc^F241A^ and Fc^Abdeg^ exhibit combinatorial antiinflammatory activity, underscoring the therapeutic potential of engineered IgG1 Fcs.

## Results

### Glycoengineering Fc^F241A^ produced in CHO-K1 cells.

As CHO-K1 cells are the preferred production line for FDA-approved biologics (51), and previous studies generated Fc^F241A^ in HEK293T cells, we asked whether CHO-K1–generated Fc^F241A^ exhibited antiinflammatory activity consistent with that produced in HEK293T cells. Wild-type human IgG1 Fc (Fc^WT^) and Fc^F241A^ (Figure 1A) were generated, and the associated glycoforms at N297 (Figure 1B) were analyzed by high-performance liquid chromatography (HPLC) (Figure 1C). While N297 on Fc^WT^ was predominantly occupied by fucosylated agalactosylated or monogalactosylated glycans, consistent with published data, N297 on Fc^F241A^ was occupied by a mixture of fucosylated, digalactosylated, and mono- or disialylated glycans. In total, approximately 40% of N-linked glycans on Fc^F241A^ were sialylated.

Because CHO-K1 cells attach sialic acid to galactose exclusively in α2,3 linkages, we next sought to engineer CHO-K1 cells that would attach α2,6-linked sialic acid like that found endogenously on human IgG1 (52, 53). To this end, 4 stable cultures of CHO-K1 cells that expressed human N-linked glycosylation pathway enzymes were established and used to produce Fc^F241A^. These included Fc^F241A^ generated in unmanipulated CHO-K1 cells (Fc^F241A^), Fc^F241A^ produced in cells expressing the α2,6-sialyltransferase ST6GAL1 (Fc^F241A/ST6^), Fc^F241A^ produced in cells expressing the β1,4-galactosyltransferase B4GALT1 alongside ST6GAL1 (Fc^F241A/B4ST6^) (54), and Fc^F241A^ produced in cells expressing small interfering RNA (siRNA) against the Golgi apparatus sialic acid transporter SLC35A1 (Fc^F241A/siSLC^) (55). The glycans at N297 on the resulting Fc^F241A^s were assessed by HPLC, which revealed that approximately 40% of glycans on Fc^F241A^ were terminally sialylated and sialic acid residues were primarily α2,3-linked. Glycans on Fc^F241A/ST6^ were approximately 65% terminally sialylated with a mixture of mono- and disialylation and exclusively α2,6 linkages. Glycans on Fc^F241A/B4ST6^ were about 90% di-2,6-sialylated, and finally, glycans on Fc^F241A/siSLC^ were completely asialylated and mostly digalactosylated (Figure 1D).

### Sialylation does not influence Fc^F241A^ antiinflammatory activity but increases half-life and bioavailability in vivo.

Next, we extended our studies to the K/BxN serum transfer arthritis model (Supplemental Figure 1A; supplemental material available online with this article; https://doi.org/10.1172/JCI172980DS1). K/BxN mice spontaneously develop swollen joints, and transfer of K/BxN serum leads to autoantibody-induced joint inflammation in most mouse strains (56). Importantly, high-dose IVIG and sialylated Fc^WT^ have been shown to suppress K/BxN-induced inflammation, providing a useful model to examine and compare the antiinflammatory activities of different IgGs in vivo (26, 27, 31, 32, 57). To this end, Fc^F241A^ at doses ranging from 20 to 100 mg/kg and K/BxN serum were administered to female C57BL/6 mice, and then paw swelling was monitored over the next 10 days (Supplemental Figure 2, A and B). Throughout the 10-day monitoring period and at the peak of inflammation at day 7, both the 50 and 100 mg/kg doses offered significant protection against autoantibody-induced inflammation compared with the PBS-treated group. The lower doses of 30 and 40 mg/kg showed minor but not significant protection, and the lowest dose of 20 mg/kg provided no protection. These results confirm that CHO-K1 cell–produced Fc^F241A^ exhibits antiinflammatory activity at 50 mg/kg in the K/BxN transfer model, and thus we chose 50 mg/kg as our working dose in the K/BxN model. This is a 10- to 20-fold lower dose than antiinflammatory 1 g/kg IVIG, but equimolar dosing to the 5%–10% sialylated IgG1 content of high-dose IVIG (26).

**Figure 2:**
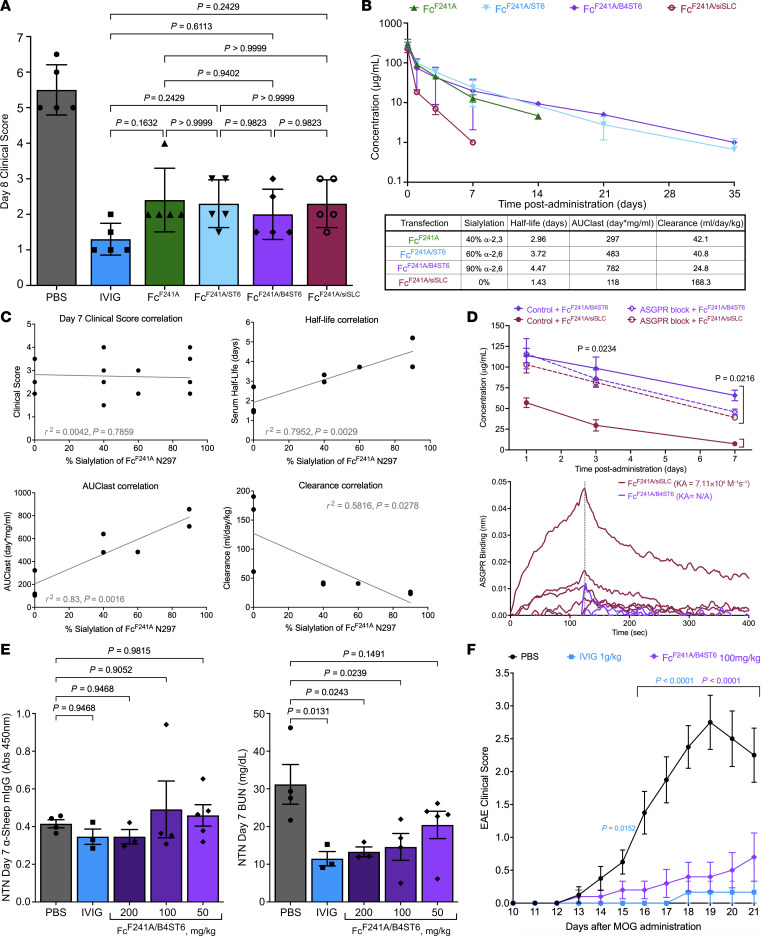
Sialylation of Fc^F241A^ is not necessary for antiinflammatory activity but improves half-life and bioavailability in vivo. (**A**) Female WT C57BL/6 mice (*n* = 5) were given K/BxN serum alongside PBS, IVIG, Fc^F241A^, Fc^F241A/ST6^, Fc^F241A/B4ST6^, or Fc^F241A/SLC35A1^ in a preventative manner, and swelling was scored for 10 days. Day 8 clinical scores, representing maximum separation between PBS and Fc^F241A^-based treatments, are plotted. (**B**) Female humanized FcRn mice (*n* = 6) were dosed with 20 mg/kg of Fc^F241A^, Fc^F241A/ST6^, Fc^F241A/B4ST6^, or Fc^F241A/siSLC^. Serum concentration of hIgG Fc was measured via ELISA. Half-life, area under the curve from the time of dosing to the last measurable concentration (AUC_last_), and clearance rate were calculated. (**C**) Correlation between sialylation on Fc^F241A^ and clinical scores, half-life, AUC_last_, and clearance. Corresponding *R*^2^ and *P* values are shown. Clinical score correlation was generated from **A**; other correlations were generated from **B**. (**D**) Female WT mice (*n* = 4) were given control or ASGPR block before Fc^F241A/B4ST6^ or Fc^F241A/siSLC^. Serum concentration of hIgG Fc was measured via ELISA at 1, 3, and 7 days. Fc^F241A/B4ST6^ and Fc^F241A/siSLC^ were also analyzed for ASGPR binding on Octet. (**E**) NTN was induced in female WT mice (*n* = 3–5) that were given PBS, IVIG, or varying doses of Fc^F241A^. Day 7 serum was used to quantify anti-sheep mIgG via ELISA, represented as absorbance at 450 nm. Day 7 blood urea nitrogen (BUN) levels for NTN mice were also quantified. (**F**) Female WT C57BL/6 mice (*n* = 6–10) had EAE induced and were treated with PBS, IVIG, or 100 mg/kg Fc^F241A^ on days 5, 10, 15, and 20. EAE clinical scores were recorded daily. Data are plotted as means with SDs in **A**, **B**, and **D**–**F**. Statistics are ordinary 1-way ANOVA with Tukey’s multiple comparisons (**A**, **B**, and **D**–**F**) or simple linear regression (**C**).

Differentially glycosylated Fc^F241A^ from each of the 4 preparations was tested at the working concentration of 50 mg/kg in the K/BxN model for its ability to prevent joint inflammation (Figure 2A and Supplemental Figure 3). All 4 glycoforms of Fc^F241A^ protected equivalently throughout the 10-day time course, regardless of sialylation status. Previous studies have established a narrow therapeutic window of the initial 2 days for IVIG to suppress K/BxN inflammation (57, 58). Additionally, the average clinical score of each Fc^F241A^-treated group was not significantly different from that of the IVIG-treated group at the peak of score separation on day 8. Thus, although sialylation in α2,6 linkages is required for the antiinflammatory activity of sialylated IgG Fcs, sialylation is not required for Fc^F241A^ to be immunomodulatory. And although not significant, after day 7 an accelerated drop in clinical score was observed in the group treated with Fc^F241A/B4ST6^ compared with those treated with the other preparations (Supplemental Figure 3), which suggested potential pharmacokinetic properties of the 90% di-2,6-sialylated glycoform of Fc^F241A^.

Full-length IgGs have an extended serum half-life of up to 21 days due to interactions with FcRn, which recycles IgG back into the circulation after cellular uptake (15). In contrast, Fc fragments have reduced serum half-life, and terminal sialylation of other glycoproteins is thought to enhance serum half-life by preventing binding with the hepatic asialoglycoprotein receptor (ASGPR) (59). Therefore, we asked whether sialylation of Fc^F241A^ impacted its serum half-life. We explored this by administering a single i.v. 20 mg/kg dose of each of the different Fc^F241A^ glycoforms to humanized FcRn Tg32 (hFcRn) mice and quantifying the amount of human IgG1 Fc in circulation over time (Figure 2B).

Following administration, Fc^F241A/siSLC^ was quickly cleared from circulation and was undetectable after 7 days, corresponding to a half-life of 1.43 days. This preparation had an area under the plasma concentration–time curve to the last measurable plasma concentration (AUC_last_) of 118 d*mg/mL, and a clearance rate of 168.3 mL/d/kg. Fc^F241A^ had a half-life of 2.96 days, AUC_last_ of 297 d*mg/mL, and clearance of 42.1 mL/d/kg. Fc^F241A/ST6^ had a 3.72-day serum half-life, a 483-d*mg/mL AUC_last_, and a clearance of 40.8 mL/d/kg. However, Fc^F241A/B4ST6^ exhibited the longest residency in the serum with a half-life of 4.47 days, and only fell below a concentration of 1 μg/mL past day 35 post-administration, with an AUC_last_ of 782 d*mg/mL and clearance rate of 24.8 mL/d/kg. Although percentage sialylation of the N297 glycan on Fc^F241A^ had no correlation with antiinflammatory activity (*R*^2^ = 0.0042), there was a significant positive correlation with half-life (*R*^2^ = 0.7952) and AUC_last_ (*R*^2^ = 0.83) and a significant negative correlation with clearance rate (*R*^2^ = 0.5816) (Figure 2C). As sialylation appeared to influence Fc^F241A^ serum half-life, we asked whether the asialoglycoprotein receptor ASGPR might accelerate clearance of asialylated Fc^F241A^. WT mice were given Fc^F241A/B4ST6^ or Fc^F241A/siSLC^ in combination with ASGPR blockade or control (Figure 2D and Supplemental Figure 4). Briefly, administration of neuraminidase-treated α1-acid glycoprotein (AGP) saturates the ASGPR. Serum half-life of Fc^F241A/B4ST6^ was unaffected by this treatment. However, Fc^F241A/siSLC^ half-life was significantly extended, and similar to that of Fc^F241A/B4ST6^, with ASGPR blockade. Indeed, biolayer interferometry studies revealed a weak interaction of Fc^F241A/siSLC^ and ASGPR, while Fc^F241A/B4ST6^ did not bind ASGPR (Figure 2D).

Based on these results, the Fc^F241A/B4ST6^ preparation with approximately 90% α2,6-sialylation on the N297 glycan was selected for use in further analyses and experiments. We next asked whether Fc^F241A/B4ST6^ possessed antiinflammatory properties using the nephrotoxic nephritis (NTN) mouse model of Goodpasture’s syndrome. Briefly, mice are immunized with sheep IgG, and develop a robust anti-sheep IgG response. These mice are then challenged with sheep anti–glomerular basement membrane (α-GBM) serum, which targets sheep:mouse IgG immune complexes to the kidneys, leading to kidney destruction (Supplemental Figure 1B). NTN-treated mice were given PBS, IVIG, or Fc^F241A/B4ST6^ at 200 mg/kg, 100 mg/kg, or 50 mg/kg. Anti-sheep IgG titers were similar across all treatment groups, indicating that IVIG and Fc^F241A^ did not impact the pathogenic immune response (Figure 2E). Blood urea nitrogen levels at 7 days were elevated in PBS-treated animals, but significantly reduced by IVIG and Fc^F241A^ at 200 mg/kg and 100 mg/kg, but not 50 mg/kg (Figure 2E).

Next, the antiinflammatory activity of Fc^F241A^ was examined in experimental autoimmune encephalomyelitis, a mouse model of multiple sclerosis (Supplemental Figure 1C). Mice were immunized with myelin oligodendrocyte glycoprotein (MOG) and treated with PBS, IVIG (1 g/kg), or Fc^F241A/B4ST6^ at 100 mg/kg. While PBS treatment resulted in severe disease, both IVIG and Fc^F241A/B4ST6^ significantly attenuated disease (Figure 2F), consistent with previous results (34).

### Preferential receptor engagement distinguishes Fc^F241A/B4ST6^ and Fc^Abdeg^.

**Figure 3:**
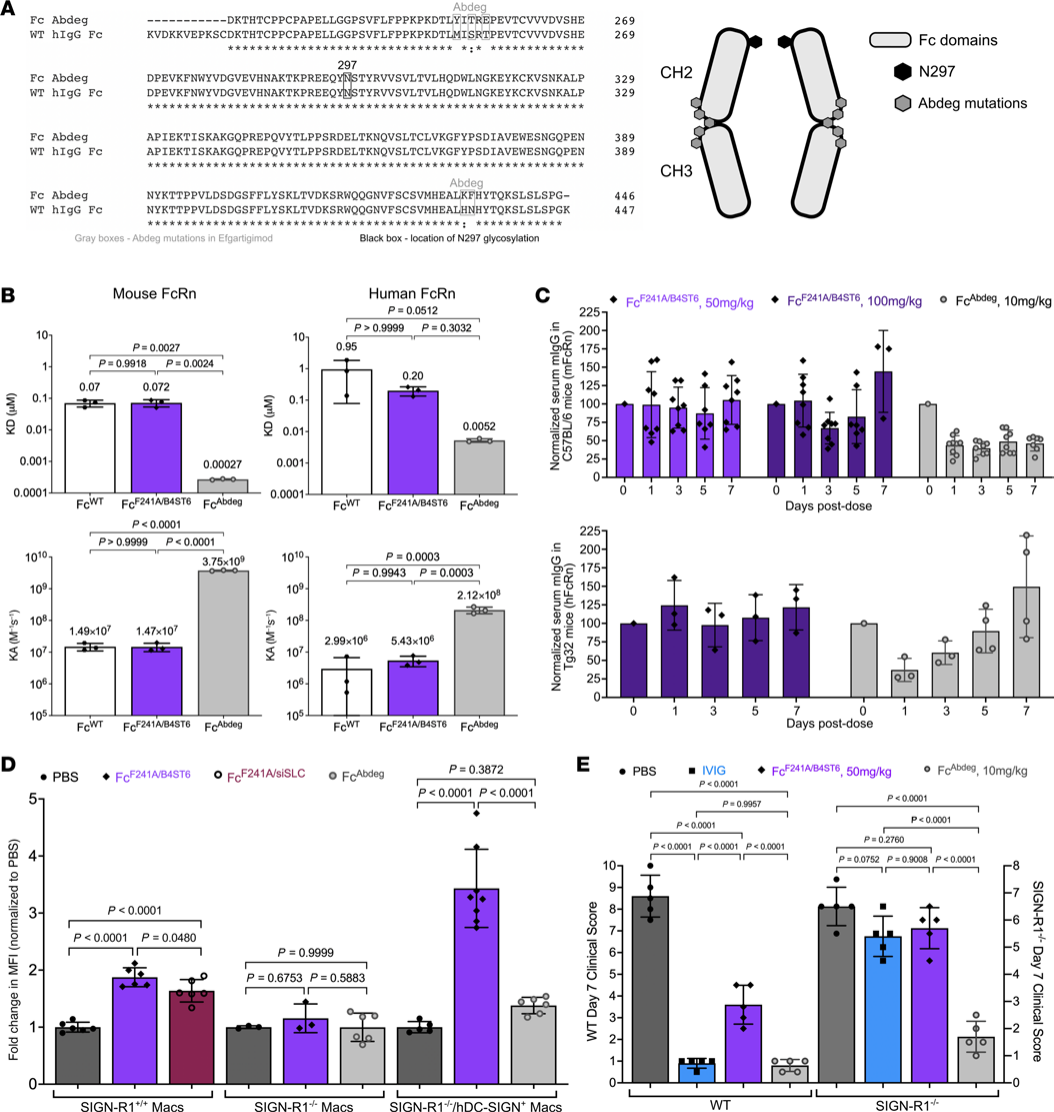
Divergent antiinflammatory pathways are elicited by Fc^F241A/B4ST6^ and Fc^Abdeg^. (**A**) Sequence alignment and schematics of Fc^WT^ and Fc^Abdeg^, with the gray boxes designating the locations of Abdeg mutations and the black box designating the location of N297. (**B**) Dissociation constants (*K_D_*, μM) and association constants (*K_A_*, M^–1^ s^–1^) determined by surface plasmon resonance (SPR) of Fc^WT^, Fc^F241A/B4ST6^, and Fc^Abdeg^ with mFcRn and hFcRn are plotted. (**C**) Female WT C57BL/6 (*n* = 3–9) and humanized homozygous FcRn (Tg32) mice (*n* = 3–4) were given 1 dose of 50 or 100 mg/kg of Fc^F241A/B4ST6^ or 10 mg/kg of Fc^Abdeg^, and serum mouse IgG was measured via ELISA out to day 7 post-dose. (**D**) Fold change of cell surface binding MFI of PBS, Fc^F241A/B4ST6^, Fc^F241A/siSLC^, or Fc^Abdeg^ to SIGN-R1^+/+^, SIGN-R1^–/–^, and hDC-SIGN^+^/SIGN-R1^–/–^ murine bone marrow–derived macrophages (BMDMs) as detected by FACS. Plot shows the fold change in binding, represented as MFI, in comparison with PBS. (**E**) Female WT C57BL/6 mice (*n* = 5 per group) and female and male SIGN-R1^–/–^ mice (*n* = 5) were given arthritogenic K/BxN serum alongside PBS, IVIG 1 g/kg, Fc^F241A/B4ST6^ 50 mg/kg, or Fc^Abdeg^ 10 mg/kg in a preventative manner, and joint swelling was clinically scored for 10 days. Day 7 clinical scores for each group, representing the maximum separation in clinical score between PBS and Fc^F241A^-based treatments, are plotted. Bar graphs are plotted as means with SDs, and statistics are ordinary 1-way ANOVA with Tukey’s multiple comparisons in **B**, **D**, and **E**.

Fc^Abdeg^ (efgartigimod) is a recently FDA-approved therapeutic that accelerates the depletion of circulating total IgG by possessing enhanced affinity for and saturating FcRn and is currently approved to treat myasthenia gravis (44). Fc^Abdeg^ contains Abdeg mutations in the region of the Fc that binds to FcRn and thus enables it to outcompete native IgG (Figure 3A) (43, 44, 49). Following our findings of Fc^F241A/B4ST6^ sialylation enhancing half-life, we sought to confirm the pathways and requirements for the antiinflammatory activity of Fc^Abdeg^ and Fc^F241A/B4ST6^.

We first examined the interactions of Fc^WT^, Fc^F241A/B4ST6^, and Fc^Abdeg^ with mouse and human FcRn by biolayer interferometry (Figure 3B and Supplemental Figure 5). Fc^WT^ and Fc^F241A/B4ST6^ bound similarly to mouse and human FcRn. In contrast, the dissociation constant (*K_D_*) of Fc^Abdeg^ was 267-fold and 39-fold lower than that of Fc^F241A/B4ST6^ to mouse FcRn and human FcRn, respectively. Subsequently, we examined the effects of both mutant Fcs on circulating mouse IgGs through FcRn (Figure 3C). C57BL/6 and hFcRn (Tg32) mice were given a single i.v. injection of Fc^F241A/B4ST6^ or Fc^Abdeg^, and after dosing, serum total mIgG was determined via ELISA. In WT C57BL/6 mice with murine FcRn, Fc^F241A/B4ST6^ treatment did not reduce serum IgG titers. In contrast, Fc^Abdeg^ induced a marked immediate and lasting reduction in IgG titers, consistent with the greatly enhanced affinity of Fc^Abdeg^ for murine FcRn relative to Fc^F241A/B4ST6^. Furthermore, Fc^F241A/B4ST6^ did not impact IgG serum titers in hFcRn (Tg32) mice. However, Fc^Abdeg^ induced a transient drop in IgG titers on day 1. These results are consistent with the remarkably high affinity of Fc^Abdeg^ for mFcRn, and relatively lower affinity for hFcRn, requiring multiple doses in humans to trigger long-term reductions in serum IgG.

Previous studies have demonstrated that IVIG and sialylated IgG Fc require the murine C-type lectin specific ICAM-3–grabbing non-integrin-related-1 (SIGN-R1; CD209b) to mediate antiinflammatory activity in vivo (35, 60). This requirement can be circumvented by introduction of SIGN-R1’s human ortholog, dendritic cell–specific ICAM-3–grabbing non-integrin (DC-SIGN; CD209) (61). Therefore, we examined the abilities of both Fc^F241A/B4ST6^ and Fc^Abdeg^ to bind SIGN-R1 and DC-SIGN via a cell-binding assay (Figure 3D). We isolated and immortalized bone marrow–derived macrophages (BMDMs) from SIGN-R1^+/+^, SIGN-R1^–/–^, and hDC-SIGN^+^/SIGN-R1^–/–^ mice (31, 62, 63), blocked type I FcγRs, and incubated the cells with PBS, Fc^F241A/siSLC^, Fc^F241A/B4ST6^, or Fc^Abdeg^. The BMDMs were analyzed by flow cytometry to detect surface-bound IgG. Both Fc^F241A/B4ST6^ and Fc^F241A/siSLC^ bound to SIGN-R1^+/+^ BMDMs, indicating that sialylation of Fc^F241A^ is not required for type II Fc receptor binding. Indeed, no binding was detected on the SIGN-R1^–/–^ BMDMs incubated with Fc^F241A/B4ST6^ or Fc^Abdeg^. However, binding was detected on hDC-SIGN^+^/SIGN-R1^–/–^ BMDMs treated with Fc^F241A/B4ST6^, but not with Fc^Abdeg^. Similarly, Fc^F241A/B4ST6^ was determined to bind SIGN-R1^+/+^, but not SIGN-R1^–/–^, peritoneal macrophages freshly isolated from mice (Supplemental Figure 6).

For in vivo confirmation of mechanistic differences, we administered PBS, high-dose IVIG, Fc^F241A/B4ST6^, or Fc^Abdeg^ followed by K/BxN sera to C57BL/6 and SIGN-R1^–/–^ mice, and tracked paw swelling over 10 days (Figure 3E and Supplemental Figure 7). In WT mice, marked paw swelling was observed only in the PBS-treated mice, consistent with previous data. In SIGN-R1^–/–^ mice, swelling was observed in PBS-, IVIG-, and Fc^F241A/B4ST6^-treated mice, also consistent with previous results and demonstrating that the antiinflammatory activity of both IVIG and Fc^F241A^ is dependent on SIGN-R1. However, SIGN-R1^–/–^ mice given Fc^Abdeg^ had significantly less inflammation as measured by clinical score, indicating that Fc^Abdeg^ functions independently of SIGN-R1. Taken together, these results demonstrate that Fc^F241A/B4ST6^ and Fc^Abdeg^ suppress autoantibody-induced inflammation through unique receptors and pathways.

### Combinatorial antiinflammatory activity of Fc^F241A/B4ST6^ and Fc^Abdeg^.

**Figure 4:**
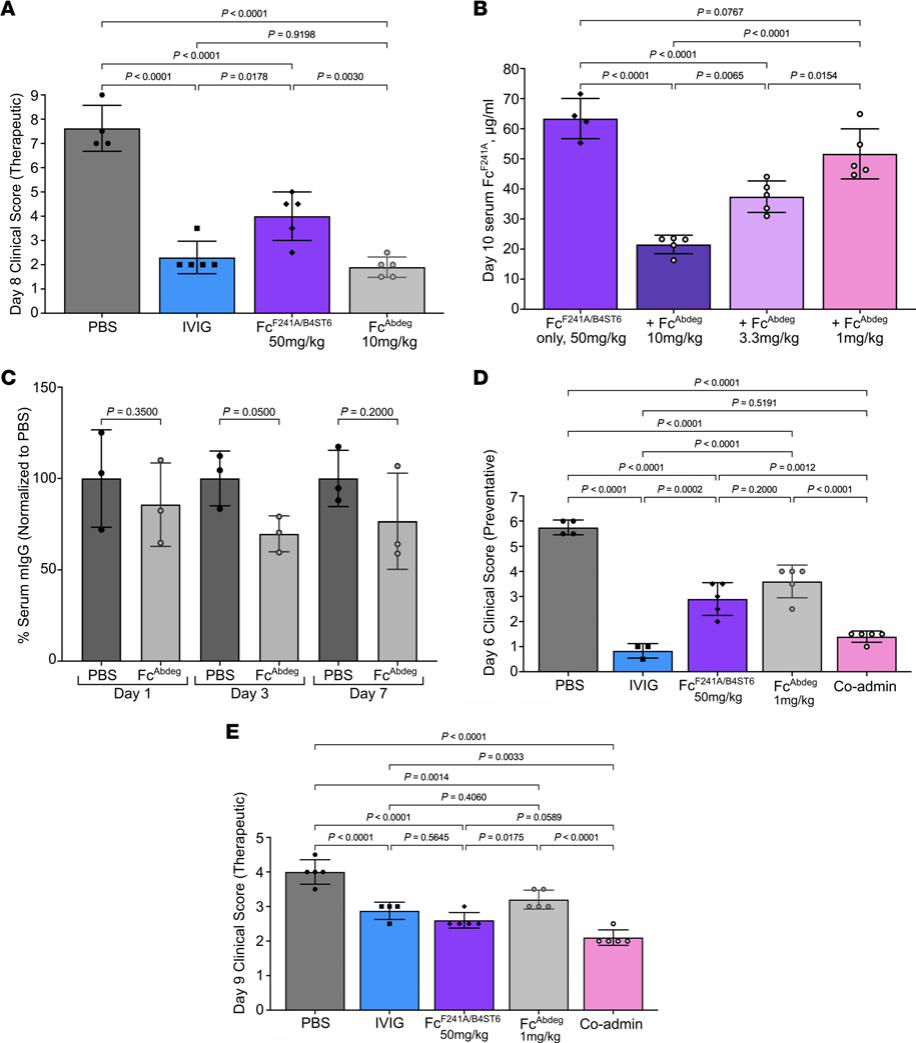
Combinatorial antiinflammatory activity of Fc^F241A/B4ST6^ and Fc^Abdeg^. (**A**) Day 8 clinical scores of mice (*n* = 5) given arthritogenic K/BxN serum on day 0, then PBS, IVIG 1 g/kg, Fc^F241A/B4ST6^ 50 mg/kg, or Fc^Abdeg^ 10 mg/kg on day 2. (**B**) Day 10 serum concentrations of Fc^F241A/B4ST6^ in mice (*n* = 4–5) given Fc^F241A/B4ST6^ 50 mg/kg alone or combined with increasing doses of Fc^Abdeg^ (1–10 mg/kg). (**C**) Relative serum IgG titers of mice (*n* = 3) given PBS or Fc^Abdeg^ 1 mg/kg, then serum mouse IgG levels days 1, 3, and 7 after administration. Data are normalized to PBS (100%). (**D**) Day 6 clinical scores of K/BxN-treated mice (*n* = 3–5) given PBS, IVIG 1 g/kg, Fc^F241A/B4ST6^ 50 mg/kg, Fc^Abdeg^ 1 mg/kg, or a combination of Fc^F241A/B4ST6^ and Fc^Abdeg^. (**E**) Day 9 clinical scores of K/BxN-treated mice (*n* = 5) given PBS, IVIG 1 g/kg, Fc^F241A/B4ST6^ 50 mg/kg, Fc^Abdeg^ 1 mg/kg, or a combination of Fc^F241A/B4ST6^ and Fc^Abdeg^ on day 2. All bar graphs are plotted as means with SDs. Statistics are ordinary 1-way ANOVA with Tukey’s multiple comparisons (**A**, **B**, **D**, and **E**) or 1-tailed Mann-Whitney test (**C**).

We next asked whether the two Fc mutants would work to effectively attenuate autoantibody-induced inflammation when administered together. To answer this question, we compared the behavior of Fc^F241A/B4ST6^ and Fc^Abdeg^ in the K/BxN model in both a preventative (Figure 3E and Supplemental Figure 7) and a therapeutic (Figure 4A, Supplemental Figure 1D, and Supplemental Figure 8) manner. Both mutant Fcs significantly protected against inflammation in a manner mostly comparable to that of high-dose IVIG in both treatment modalities.

As Fc^Abdeg^ suppresses IgG-mediated inflammation by saturating FcRn, thereby reducing total IgG titers, we reasoned that Fc^Abdeg^ might also lead to more rapid clearance of Fc^F241A/B4ST6^. To determine a dose combination at which the clearance effects of Fc^Abdeg^ would not interfere with the activity or half-life of Fc^F241A/B4ST6^, we administered 50 mg/kg of Fc^F241A/B4ST6^ in combination with decreasing doses of Fc^Abdeg^ to C57BL/6 mice and measured human Fc titers at day 10 post-administration (Figure 4B). Fc^Abdeg^ caused a decrease in circulating Fc^F241A/B4ST6^ in a dose-dependent manner as measured by ELISA. However, Fc^Abdeg^ at 1 mg/kg, one-tenth of the clinical dose, induced minimal clearance of circulating Fc^F241A/B4ST6^ and a small decrease in total serum mouse IgG consistently over 7 days, with a significant decrease achieved only on day 3 (Figure 4C).

Because Fc^Abdeg^ displays at least a 10-fold enhanced affinity for mouse FcRn compared with human FcRn, we reasoned that 10-fold lower dosing would be more representative of the human system and would not deplete Fc^F241A/B4ST6^ titers. Thus, C57BL/6 mice were given PBS, high-dose IVIG, 50 mg/kg Fc^F241A/B4ST6^, 1 mg/kg Fc^Abdeg^, or 50 mg/kg Fc^F241A/B4ST6^ coadministered with 1 mg/kg of Fc^Abdeg^ in both the preventative and the therapeutic K/BxN model. In the preventative model, IVIG, 50 mg/kg Fc^F241A/B4ST6^, and 1 mg/kg of Fc^Abdeg^ all effectively reduced inflammation in comparison with PBS-treated mice (Figure 4D and Supplemental Figure 9). However, mice that received the coadministered Fc^F241A/B4ST6^ and Fc^Abdeg^ exhibited even more reduced inflammation.

We next tested the potential of combinatorial antiinflammatory biologicals in the therapeutic K/BxN model (Figure 4E and Supplemental Figure 10). As above, mice were given arthritogenic K/BxN sera on day 0, and PBS, high-dose IVIG, 50 mg/kg Fc^F241A/B4ST6^, 1 mg/kg of Fc^Abdeg^, or 50 mg/kg Fc^F241A/B4ST6^ and 1 mg/kg of Fc^Abdeg^ on day 2, and foot swelling was monitored over 12 days. The coadministration treatment dampened inflammation by day 6, 4 days after treatment. This was significantly different from the IVIG and Fc^Abdeg^-alone treatments. These data suggest that Fc^F241A/B4ST6^ and Fc^Abdeg^ function through unique pathways and receptors that complement one another in vivo.

## Discussion

Treatment of patients suffering from autoimmune and inflammatory diseases with high-dose IVIG, although effective, is often not well tolerated because of the large protein dosages required for antiinflammatory activity. Indeed, infusion often occurs monthly, and can be prohibitively expensive. Further, a recent worldwide shortage of IVIG restricted use and led to recommendations of dose reductions, despite the well-known dose dependence for antiinflammatory activity. Thus, there is a need for an alternative therapeutic that mimics the antiinflammatory mechanisms of IVIG that is more cost-effective and more easily administered and can be made recombinantly in large quantities (64–66).

Here, we extensively characterized the antiinflammatory activity of recombinant mutant IgG Fc^F241A^ in vivo. We confirmed that the antiinflammatory activity is intact in CHO-K1–generated material. These cell lines are used in industrial biotechnology to produce therapeutic IgG and other mammalian recombinant proteins. Indeed, the glycosylation profile of the single glycan on Fc^F241A^ at N297 shows enhanced sialylation compared with Fc^WT^ when produced in CHO-K1 cells. However, endogenous IgGs are terminally sialylated with α2,6 linkages, and unmodified CHO-K1 cells can only add sialic acid in α2,3 linkages. To circumvent potential antigenicity of α2,3-sialylated Fc^F241A^, we engineered a panel of CHO-K1 cell lines that added varying degrees of sialylation to Fc^F241A^, including 0% sialylation, 40% in α2,3 linkages, 60% in α2,6 linkages, and 90% in α2,6 linkages.

Intriguingly, although the sialic acid content of Fc^F241A^ had no impact on its antiinflammatory activity, increasing sialylation led to improved serum half-life and bioavailability. These results were surprising as IgG half-life is thought to be primarily regulated by FcRn (67). It is also known that the hepatic ASGPR can rapidly clear terminally galactosylated and asialylated proteins from circulation (68). Although the Fc glycan is thought to be buried in a pocket at the top of the CH2 domain and largely inaccessible, our data demonstrate that terminal sialylation of the Fc^F241A^ glycan improves half-life and bioavailability via decreased affinity for ASGPR. Thus, ASGPR-mediated clearance of Fc^F241A^ may be circumvented by increased terminal sialylation.

Finally, we compared the antiinflammatory activity of Fc^F241A^ with that of Fc^Abdeg^, which the FDA recently approved for treatment of myasthenia gravis under the trade name efgartigimod. Fc^Abdeg^ has enhanced affinity to FcRn, and reduces total IgG titers, including pathogenic autoantibodies, after repeated administration in patients. This has been shown to reduce the clinical symptoms of myasthenia gravis; however, patients treated with repeated dosing of Fc^Abdeg^ may become immunocompromised to some degree as a result of IgG depletion. In binding assays, Fc^F241A^ and Fc^WT^ bound to mouse and human FcRn similarly, while Fc^Abdeg^ bound to mouse FcRn with 267-fold enhanced affinity and to human FcRn with approximately 96-fold enhanced affinity compared with Fc^F241A^ and Fc^WT^. Importantly, Fc^F241A^ was found to bind to the antiinflammatory type II FcγRs SIGN-R1 and its human ortholog DC-SIGN, while Fc^Abdeg^ did not. In vivo, Fc^Abdeg^ triggered rapid clearance of total IgG from circulation, while Fc^F241A^ did not impact circulating IgG titers. Coadministration of Fc^Abdeg^ and Fc^F241A^ significantly inhibited joint inflammation in the K/BxN model compared with individual treatments, both of which were able to suppress arthritogenic inflammation on their own. Taken together, these results demonstrate that Fc^F241A^ and Fc^Abdeg^ exert immune protection by distinct mechanisms.

The studies reported here enhance the current knowledge on the in vitro and in vivo activities of Fc^F241A^ and Fc^Abdeg^, demonstrate that their mechanisms of action are distinct (Fc^F241A^ and IVIG exert immune protection through SIGN-R1/DC-SIGN while Fc^Abdeg^ does not), and illustrate their potential utility in the treatment of autoimmune conditions. Additional studies should be conducted with this Fc^F241A^ preparation in additional experimental and animal models to characterize its potential clinical efficacy further.

## Methods

### Production of recombinant human IgG1 Fcs.

Curia Global (San Carlos, California, USA) was contracted to produce recombinant human IgG1 Fcs with a WT sequence (Fc^WT^), IgG1 Fc bearing the F241A mutation (Fc^F241A^), and IgG1 Fc bearing the Abdeg mutations (Fc^Abdeg^). These were produced by transient transfection of CHO-K1 cells (ATCC) and purified by protein A affinity chromatography.

Four different CHO-K1 cell stable production pools were generated by stable transfection to produce 4 recombinant human IgG1 Fc^F241A^ proteins with different sialic acid content at N297. The first was generated by expression of Fc^F241A^ alone. The second was generated by stable coexpression of Fc^F241A^ and hST6GAL1 (Fc^F241A/ST6GAL1^). The third was generated by coexpression of Fc^F241A^, hST6GAL1, and hB4GALT1 (Fc^F241A/B4ST6^). The fourth was generated by coexpression of Fc^F241A^ and a small interfering RNA (siRNA) for Golgi–sialic acid transporter SLC35A1 (Fc^F241A/siSLC^). The IgG1 Fc^F241A^ proteins were purified from 1L cultures of these stable cell lines by protein A chromatography.

### Analysis of N-linked glycans.

N-linked glycan analysis for IgG Fcs was performed by ATUM. N-glycans were released and labeled using the InstantPC kit (Agilent). Briefly, 20 mg of intact Fcs and 2 mL of Gly-X denaturant were combined, incubated at 90°C for 3 minutes, and then allowed to cool at room temperature for 2 minutes. Two milliliters of N-glycanase was added to each sample, mixed, and then incubated at 50°C for 5 minutes. Five milliliters of InstantPC Dye solution was added, and samples were incubated for a further 1 minute at 50°C. To each sample, 150 mL of load/wash solution was applied. The samples were washed 3 times with load/wash buffer and vacuum-filtered each time before the labeled N-glycans were eluted with 100 mL of Gly-X InstantPC. The samples were either run immediately on an HPLC-HILIC-FLD or Agilent 6530-QTOF or stored for later analysis with a foil plate seal at –20°C.

InstantPC-labeled glycans were analyzed by HPLC-HILIC-FLD on an Agilent 1290 HPLC system with a fluorescence detector (Agilent) using an AdvanceBio Glycan mapping 300 Å column (1.8 mm, 2.1 × 150 mm, Agilent) with an increasing ammonium formate linear gradient (mobile phase A: 100 mM ammonium formate, pH 4.5, in water; mobile phase B: acetonitrile) at a flow rate of 0.6 mL/min. An injection volume of 2 μL and a column temperature of 40°C were used. Glycans were detected at a wavelength of 345 nm with an excitation wavelength of 285 nm. Peaks were integrated using OpenLabs CDS software (Agilent), and the relative glycan compositions were calculated. In conjunction with the samples being run, a dextran ladder (AdvanceBio InstantPC Maltodextrin ladder, Agilent) was run before and after the samples. The ladder was used to calibrate the HPLC runs and to plot a curve to allocate standardized glycan unit (GU) values from retention times. Calculated GU values were compared with a database of reference structures (InstantPC Labeled Glycans, Agilent), allowing glycoform identities to be assigned to each peak. The relative abundance (%Area) of each glycan is expressed as the average of the percentage of the total peak area. Some released glycan samples were analyzed by mass spectroscopy, injected onto an Agilent 6530-QTOF, and analyzed for glycans in positive mode.

### In vivo murine studies.

Nephrotoxic nephritis (NTN), K/BxN serum transfer, and ASGPR blocking animal studies were conducted under protocols approved by the Institutional Animal Care and Use Committee of Massachusetts General Hospital (MGH). Our study examined male and female animals, and similar findings are reported for both sexes. WT C57BL/6 (strain 000664) mice were purchased from The Jackson Laboratory and were 7–8 weeks old and were group-housed and maintained in the animal facility at MGH under specific pathogen–free conditions and given food and water ad libitum according to National Institutes of Health (NIH) guidelines.

NTN was induced by first administering 200 mg of sheep IgG (Bio-Rad PSP01) intraperitoneally in complete Freund’s adjuvant (day –4). Four days later (day 0), mice were given i.v. PBS, IVIG (Gammagard, Takeda) 1 g/kg, or Fc^F241A^ at 50–200 mg/kg. One hour later, mice received an i.v. injection of sheep α-GBM serum (Probetex) at a dose of 2 μL per gram of body weight. On day 7, serum was collected from treated mice for measurement of mouse anti-sheep IgG (see below) and blood urea nitrogen. Blood urea nitrogen was determined by the Urea Assay Kit (Stanbio) using the enzyme-coupled equilibrium method.

K/BxN serum transfer arthritis was conducted under protocols approved by the Institutional Animal Care and Use Committee of MGH. Briefly, group-housed 7- to 8-week-old female WT C57BL/6 (strain 000664) mice were purchased from The Jackson Laboratory, maintained in the animal facility at MGH under specific pathogen–free conditions, and given food and water ad libitum according to NIH guidelines. SIGN-R1^–/–^ and hDC-SIGN^+^/SIGN-R1^–/–^ mice on a C57BL/6 background were gifts from J. Ravetch (The Rockefeller University, New York, New York, USA) (31, 62, 63). KRN TCR-transgenic mice on a C57BL/6 background (K/B) were gifts from D. Mathis and C. Benoist (Harvard Medical School, Boston, Massachusetts, USA) and were bred to male NOD mice (strain 032445) purchased from The Jackson Laboratory to generate K/BxN mice (56). K/BxN mice with visible joint inflammation were bled into serum-separating tubes (BD Biosciences), and serum was separated from the blood by centrifugation, as described previously (26). For preventative models examining antiinflammatory activity, WT or SIGN-R1^–/–^ mice were i.v. given IVIG, IgG1 Fcs, or PBS, immediately followed by a single i.v. injection of 200 μL of undiluted K/BxN serum. For therapeutic models, mice were given 200 μL of undiluted K/BxN serum i.v., and given IVIG, IgG1 Fcs, or PBS i.v. 48 hours later. Arthritis was scored by clinical examination. A score of 0–3 was given to each paw, with a score of 0 representing no inflammation in the paw and a score of 3 representing severe inflammation of all joints in the paw, and the score of each paw was summed. Total score of each individual mouse in a treatment group is plotted.

For blocking of the hepatic asialoglycoprotein receptor (ASGPR), mice were given i.v. 50 mg/kg α1-acid glycoprotein (AGP; control) or neuraminidase-treated AGP (ASGPR block) 1 hour before i.v. administration of 20 mg/kg of Fc^F241A/B4ST6^ or Fc^F241A/siSLC^ (day 0). On day 3, mice were given an additional dose of AGP, or neuraminidase-treated AGP (ASGPR block). Mouse serum was collected on days 1, 3, and 7. Serum Fc^F241A/B4ST6^ or Fc^F241A/siSLC^ was measured via hIgG Fc ELISA (see below).

EAE experiments were conducted at BioDuro-Sundia under protocols approved by the Institutional Animal Care and Use Committee. Female WT C57BL/6 (strain 000664) mice were purchased from Shanghai SIPPR-Bk Laboratory Animal Co. Ltd. and were 6–7 weeks old and were group-housed and maintained in the animal facility at BioDuro-Sundia under specific pathogen–free conditions and given food and water ad libitum according to NIH guidelines. MOG_35–55_ was diluted in PBS to a final concentration of 3.0 mg/mL, and added to complete Freund’s adjuvant to a final concentration of 8.0 mg/mL. The mixture was emulsified for 1 hour on ice. Pertussis toxin was diluted in sterile water to a final concentration of 1.0 μg/mL. Mice were anesthetized with isoflurane and injected subcutaneously with MOG emulsion in 4 different regions for a total volume of 200 μL (day 0). Then, at hours 0 and 48 each mouse was given 250 μL of pertussis toxin solution intraperitoneally. Experimental treatments (PBS, IVIG 1 g/kg, and Fc^F241A^ 100 mg/kg) were administered i.v. on days 5, 10, 15, and 20. Clinical scores were recorded daily for 21 days.

Pharmacokinetic studies of Fc^F241A^ were conducted at Bayside BioSciences. Briefly, group-housed 5- to 7-week-old female WT C57BL/6 (strain 000664) or humanized FcRn Tg32 mice (strain 014565) obtained from The Jackson Laboratory were maintained in the animal facility under specific pathogen–free conditions and given food and water ad libitum according to NIH guidelines. Mice were randomly assigned to receive a single i.v. injection of 20 mg/kg of 1 glycoform of Fc^F241A^, with 6 mice per group. Blood was collected for serum isolation by retro-orbital bleeding at 0 minutes, 30 minutes, 1 day, 3 days, 7 days, 14 days, and 21 days, and at 35 days post-administration, mice were euthanized and blood collected via cardiac puncture. Depending on the exposure achieved, later time points were not plotted if values were below the limit of quantitation (1 μg/mL). Not all mice were bled at every time point, but each mouse contributed 3–4 times over the course of the study. Serum samples were stored at –80°C prior to shipping to Quintara Discovery for quantitation of concentration of Fc^F241A^ in the mouse serum using ELISA.

### ELISAs.

To determine the amount of serum mouse anti-sheep IgG in the NTN mouse experiments, ELISA plates were coated with 5 μg/mL of sheep IgG in coating buffer, next blocked with Pierce Protein-Free Blocking Buffer, and then incubated with mouse serum at a starting dilution of 1:200 with eight 2-fold serial dilutions. After washing with PBS containing 0.05% Tween-20, the plate was incubated with HRP-conjugated anti-mouse IgG Fc (Bethyl Laboratories catalog A90-131P). The plate was developed by addition of TMB substrate to each well for 5–10 minutes. The reaction was quenched by addition of sulfuric acid and read at 450 nm absorbance on a Biotek plate reader.

For the pharmacokinetics ELISA, anti–human IgG1 Fc capture (catalog 709-006-098) and detection (catalog 109-036-098) antibodies were obtained from Jackson ImmunoResearch. Coating buffer was sodium bicarbonate (Sigma-Aldrich catalog C3041-50CAP). Wash buffer consisted of PBS with 0.05% Tween-20. Block buffer consisted of PBS with 0.05% Tween-20 and 5% bovine serum albumin (BSA; 50 mg/mL). Dilution buffer consisted of PBS with 0.05% Tween-20 and 1% BSA (10 mg/mL). Ninety-six-well plates were coated overnight at 4°C without shaking with capture antibody diluted to 1 mg/mL in coat buffer. Plates were washed the next day with 3 rounds of wash buffer. Block buffer (80 mL) was added to each well and incubated for 2 hours at room temperature with gentle shaking. Plates were again washed 3 times, and standards (Fc^F241A^ and Fc^F241A/B4ST6^) were diluted in dilution buffer to 300 ng/mL, followed by 6 serial 1:3 dilutions to generate a 7-point standard concentration range. Mouse serum samples were diluted 1:1,000 or 1:10,000 in dilution buffer. Each standard or diluted serum sample was added to the 96-well plate and incubated for 2 hours at room temperature with gentle shaking. Plates were then washed as before, and human IgG1 Fc was detected by the addition of a 1:50,000 dilution of the detection antibody to the plate, which was then incubated overnight. The plate was washed, and color was developed by addition of TMB substrate to each well for 5–10 minutes. The reaction was quenched by addition of sulfuric acid and read at 450 nm absorbance on a Biotek plate reader.

To determine the effect of Fc^F241A/B4ST6^ and Fc^Abdeg^ on total circulating mouse IgG, female C57BL/6J mice received a single i.v. injection of 50 or 100 mg/kg Fc^F241A/B4ST6^ or 10 mg/kg of Fc^Abdeg^, and female Tg32 mice received a single i.v. injection of 100 mg/kg Fc^F241A/B4ST6^ or 10 mg/kg of Fc^Abdeg^. Whole blood was collected for serum separation before dosing and following treatment at 1, 3, 5, and 7 days after dosing. Serum was collected from 3 mice per time point. Serum samples were flash-frozen and shipped on dry ice to Quintara Discovery for analysis by standard sandwich ELISA to quantify total mouse IgG. Mouse serum was diluted 1:6,000, 1:48,000, and 1:84,000, depending on the time point and treatment condition, to best capture the quantitative values on the standard curve. All data that fit onto the linear range of the standard curve were plotted.

To determine the effect of Fc^Abdeg^ on depletion of Fc^F241A/B4ST6^ from mouse circulation, 96-well plates were coated overnight at 4°C with goat α-hIgG-Fc (Bethyl Labs) diluted 1:250 in 0.05 M bicarbonate buffer. The plate was then washed 3 times with PBS with Tween-20. Each well was blocked with 1% BSA in PBS for 2 hours at room temperature with shaking. The plate was washed as previously, and then mouse serum samples were diluted 20,000× in 1% BSA in PBS and added to the plate. Samples were incubated at room temperature for 2 hours with shaking, then washed as previously. Mouse α-hIgG-HRP detection antibody (Promega catalog W4031) was diluted 1:5,000 in 1% BSA in PBS, then added to the plate and incubated for 2 hours at room temperature with shaking. Following final washes, TMB substrate was added, and the plate was incubated in the dark with shaking for 30 minutes. The color development reaction was quenched with 1 M phosphoric acid, and the plate was read at 450 nm.

### Surface plasmon resonance and biolayer interferometry binding experiments.

Fc^WT^, Fc^F241A/B4ST6^, and Fc^Abdeg^ were immobilized on chips, and human and mouse FcRn (Acro Bioscience) was flown over a range of concentrations (1,600 nM to 50 nM) in 2-fold dilutions at pH 6.0 on a Biacore 3000 (GE Healthcare). Fc-captured biosensors were dipped in wells containing FcRn from each species at pH 6.0 at a series of 2-fold serial dilutions ranging from 1,600 nM to 50 nM, and binding response was measured. Biosensors were then dipped in buffer at pH 6.0 to observe dissociation of FcRn from captured Fcs. Biosensors were regenerated and reused for subsequent reagents. Human and murine FcRn was purchased from Acro Biosciences. Binding was measured using the Octet K2 system (Molecular Devices).

Histidine-tagged human asialoglycoprotein receptor 1 (ASGPR, Acro Biosystems) at a concentration of 100 nM was immobilized on an anti–penta-histidine (HIS1K) biosensor (Sartorius). A series of 2-fold serial dilutions of Fc^F241A^ and Fc^F241A/siSLC^ was prepared in Octet buffer, commencing from an initial 171 μg/mL concentration. The experimental protocol consisted of a 60-second baseline step, followed by a 120-second association step and a subsequent 300-second dissociation step. To regenerate the sensor after each cycle, it was immersed in a glycine HCl buffer (10 mM, pH 1.5) for 30 seconds. Binding was measured using the Octet K2 system.

### Immortalized bone marrow–derived macrophages.

To differentiate bone marrow–derived macrophages (BMDMs), bone marrow was flushed from the tibiae and femurs of SIGN-R1^–/–^ and hDC-SIGN^+^/SIGN-R1^–/–^ mice and allowed to adhere to a non-treated tissue culture plate for 1 day. Non-adherent cells were then differentiated into macrophages in DMEM containing 10% FBS, 1% l-glutamine, 1% penicillin/streptomycin, 0.1% β-mercaptoethanol, 5 ng/mL of interleukin-3 (mouse IL-3, PeproTech), and macrophage colony-stimulating factor (mouse M-CSF, PeproTech) for 7 days in non-treated tissue culture plates. To immortalize BMDMs, filtered supernatants from Cre-J2 cells that produce Cre-J2 retrovirus (69) were supplemented with 5 ng/mL of M-CSF and placed on the BMDMs. This was repeated with a second round of viral infection 24 hours later. Cells were then split with gradually decreasing concentrations of M-CSF over 2 months. hDC-SIGN^+^/SIGN-R1^–/–^ immortalized BMDMs were then sorted for hDC-SIGN expression by FACS. For maintenance and experiments, BMDMs were cultured in DMEM with 10% FBS and 1% penicillin/streptomycin.

### SIGN-R1 and DC-SIGN cell-binding assays.

Cell-binding assays were conducted as previously described, with slight modifications (70). SIGN-R1^+/+^ bone marrow was flushed from the tibiae and femurs of mice, and extracted cells were cultured in DMEM supplemented with 10% FBS, 1% penicillin/streptomycin, 1% l-glutamine 200 mM, 0.1% β-mercaptoethanol, and 40 ng/mL M-CSF (PeproTech) for 5 days before use. SIGN-R1^–/–^ and SIGN-R1^–/–^/DC-SIGN^+^ cells were immortalized murine BMDMs (see above). Cells were cultured in high-glucose DMEM (Gibco) supplemented with 1× Antibiotic-Antimycotic (Gibco) and 1× FBS (Cytiva) at 37°C and 5% CO_2_. Cells were harvested and resuspended in DC-SIGN binding buffer (1× TBS, 1 nM CaCl_2_, 2.5% FBS, and 0.05% sodium azide). Fc receptors were blocked by addition of TruStainFcX (anti–mouse CD16/32) antibody clone 93 (BioLegend 101320) and anti–mouse CD16.2 FcγRIV antibody clone 9E9 (Bio X Cell) for 20 minutes on ice according to the manufacturer’s instructions. After blocking, cells were washed in binding buffer, and then 100 μg/mL of Fcs were added to the cells, which were then incubated for 1 hour on ice. Afterward, cells were washed again with binding buffer. Staining for flow cytometry was performed using FITC-F4/80 (clone BM8, BioLegend), PE–anti–mouse CD11b (clone M1/70, BioLegend), and APC–anti-human IgG (clone M1310G05, BioLegend). Staining was performed according to the manufacturer’s instructions, on ice, for 30 minutes. Cells were immediately washed and resuspended in binding buffer and then analyzed on a Beckman Coulter CytoFLEX LS cytometer. Gating was performed on FlowJo (BD Biosciences). After gating on single cells, macrophages were gated as being double positive for F4/80 and CD11b. This population was then gated into histograms positive for the detection of APC-labeled antibody. Staining for DC-SIGN using an Alexa Fluor 647–anti-hCD209 (clone 9E9A8, BioLegend catalog 330106) confirmed the presence of the receptor by flow cytometry on DC-SIGN^+^ BMDMs.

### Statistics.

For all bar graphs, individual data points (from multiple replicates or individual mice) are shown, alongside means with standard deviations. Line graphs show means with standard deviations. Linear correlation plots were generated by a simple linear regression. All *P* values were calculated using an ordinary 1-way ANOVA with Tukey’s multiple comparisons except those in Figure 4C, which were calculated using a 1-tailed Mann-Whitney test. *P* values of less than 0.05 were considered significant. All plots and statistics were generated using GraphPad Prism 9 (Dotmatics).

### Study approval.

Mouse experiments performed at MGH were approved by the Institutional Animal Care and Use Committee (Animal Welfare Assurance no. A3596-01). Mouse experiments performed at Bayside BioSciences and at BioDuro-Sundia were approved by the Institutional Animal Care and Use Committees at these facilities respectively.

### Data availability.

Primary data sets are available upon request from RMA. Data sets are also available in the Supporting Data Values file.

## Author contributions

GPC, PBC, and RMA conceptualized and acquired funding for the project. GPC and PBC generated the recombinant Fcs. SLS, BBR, and SR performed in vivo experiments. AFSL and BBR performed cell-binding experiments. GPC and BBR performed biolayer interferometry experiments. GPC performed pharmacokinetics and pharmacodynamics experiments. IF and KLJ generated immortalized bone marrow–derived macrophages. SLS, BBR, AFSL, and GPC analyzed data and generated visualizations. SLS, GPC, PBC, and RMA wrote the manuscript. All authors reviewed and edited the manuscript, and approved the final version as submitted.

## Supplementary Material

Supplemental data

Supporting data values

## Version 1 02/15/2024

Electronic publication
