# Supplementary material for: An engineered immunomodulatory IgG1 Fc suppresses autoimmune inflammation through pathways shared with i.v. immunoglobulin
Source: J Clin Invest. 2024 Feb 15;134(4):e172980. doi: 10.1172/JCI172980 (PMC10866649; doi:10.1172/JCI172980)
Supplement: Supplemental data [file jci-134-172980-s016.pdf]

Supporting Data for

An Engineered Immunomodulatory IgG1 Fc Suppresses Autoimmune Inflammation Through Pathways Shared With IVIG

Sunny L. Sneed[1o], Brian B. Reese[1], Ana F.S. Laureano[1], Sneha Ratnapriya[1], Isabella Fraschilla[2+], Kate L. Jeffrey[2#], Greg P. Coffey[3], Pamela B. Conley[3], and Robert M. Anthony[1*]

[1]Center for Immunology and Inflammatory Diseases, Massachusetts General Hospital, Harvard Medical School, Boston, MA 02129.
[2]Center for the Study of Inflammatory Bowel Disease, Department of Medicine, Massachusetts General Hospital, Harvard Medical School, Boston, MA 02114.
[3]Nuvig Therapeutics, 1775 Woodside Road, Suite 201, Redwood City, CA 94061, USA.
[o]Current address: Department of Microbiology and Immunology, Brody School of Medicine, East Carolina University, Greenville, NC 27834, USA.
[+]Current address: Koch Institute for Integrative Cancer Research at MIT, 77 Massachusetts Avenue; 76-453, Cambridge, MA 02139, USA.
[#]Current address: Moderna Therapeutics, 200 Technology Square, Cambridge, MA 02139, USA.

***Corresponding author:**

Robert M. Anthony

Email: robert.anthony@mgh.harvard.edu

Phone: 617-724-4245

Massachusetts General Hospital, Harvard Medical School, Department of Medicine, Division of Rheumatology, Allergy, and Immunology, Center for Immunology and Inflammatory Diseases, 149 13th Street, Boston, MA 02129, USA.

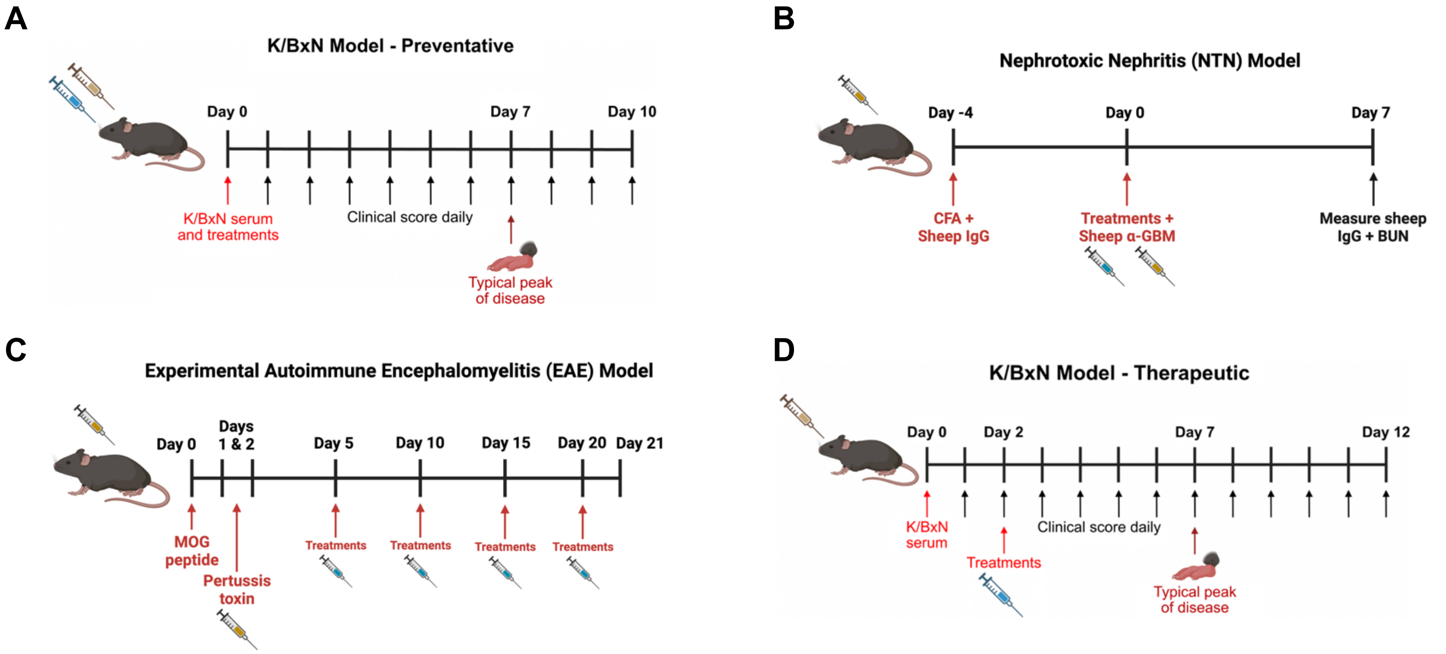

Figure S1. (A) Experimental timeline for the preventative K/BxN mouse model. **(B)** Experimental timeline for the nephrotoxic nephritis (NTN) mouse model. **(C)** Experimental timeline for the experimental autoimmune encephalomyelitis (EAE) mouse model. **(D)** Experimental timeline for the therapeutic K/BxN mouse model.

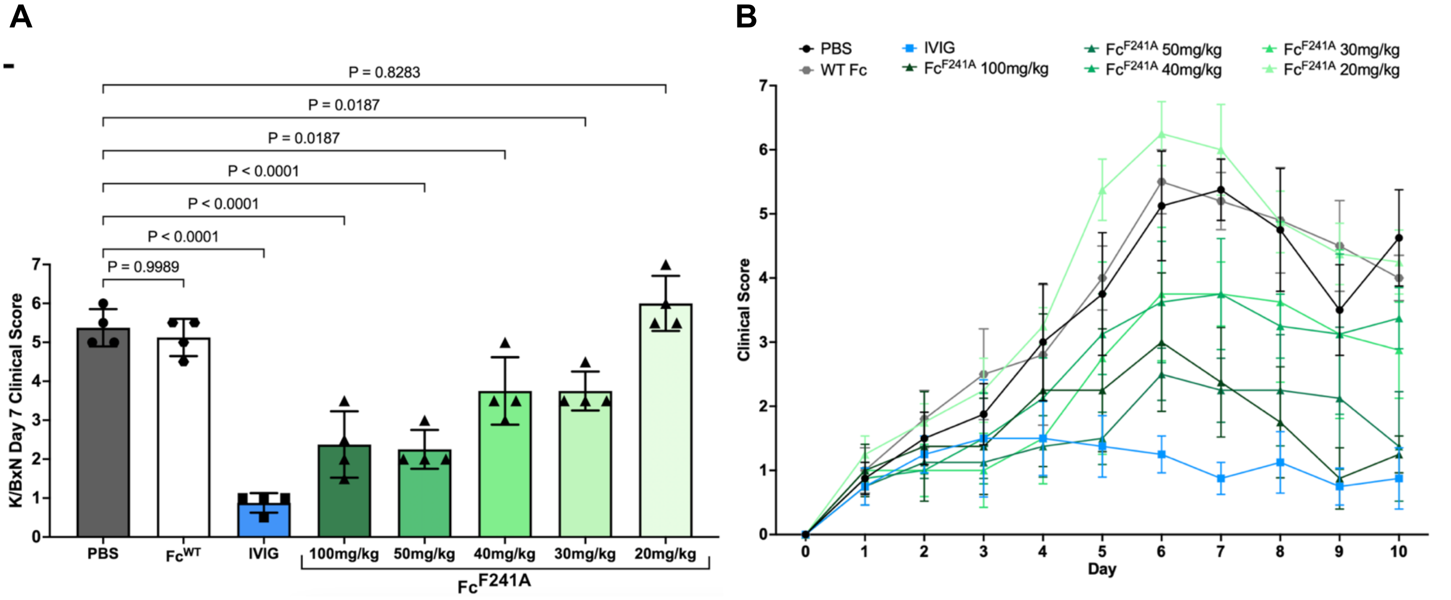

Figure S2. (A) Day 7 clinical scores of K/BxN serum treated-female WT C57BL/6 mice (N=4) administered PBS, Fc^{WT}, IVIG, or varying doses of Fc^{F241A} (20-100mg/kg). **(B)** Full time course of the experiment. Mice were administered PBS (black circles), WT asialylated IgG Fc (gray hexagons), IVIG (blue squares), or various dosages of Fc^{F241A} (green triangles) alongside K/BxN serum on day 0. Clinical scores of joint inflammation were recorded for 10 days.

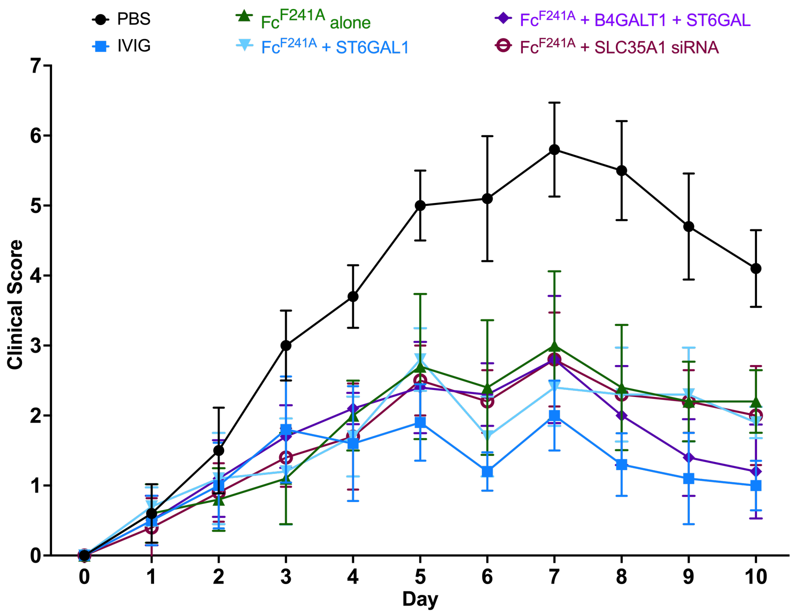

Figure S3. Full time course of K/BxN experiment shown in main text Figure 2A. Female WT C57BL/6 mice (N=5 per group) were administered PBS (black circles), IVIG (blue squares), or 50mg/kg of body weight of the differeing glycoforms of Fc^{F241A} alongside K/BxN serum on day 0. Clinical scores of joint inflammation were recorded for 10 days.

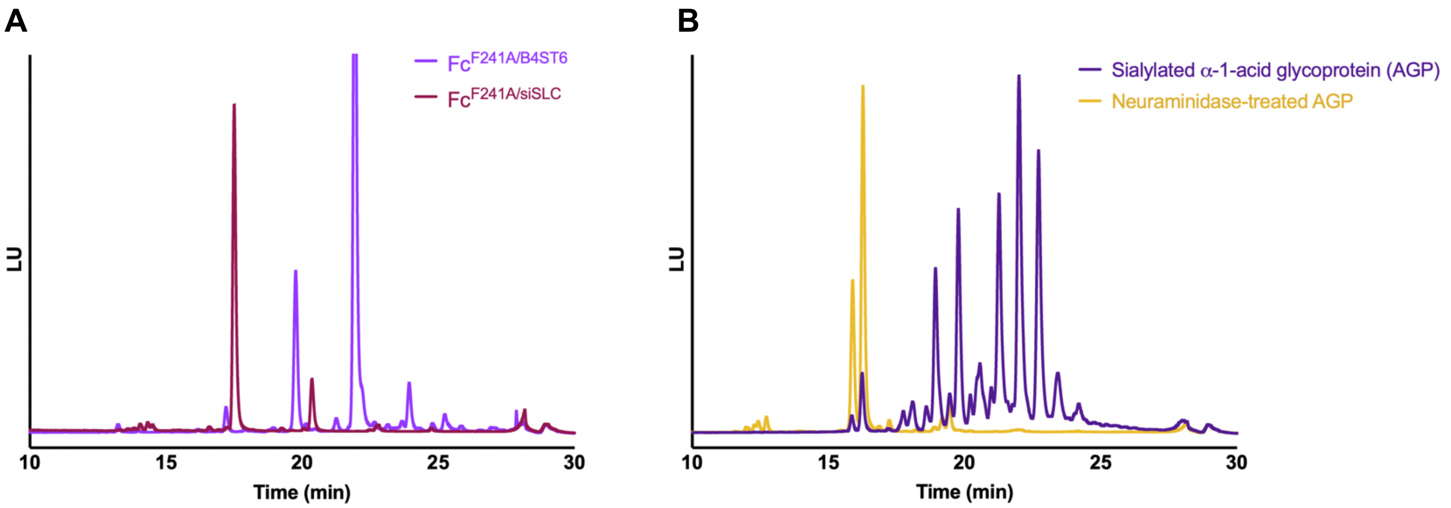

Figure S4. Representative HPLC traces for reagents used in main text Figure 2E, with luminescence in arbitrary units on the Y-axis. **(A)** Traces for $Fc^{F241A/B4ST6}$ and $Fc^{F241A/siSLC}$. **(B)** Traces for sialylated α-1-acid glycoprotein (AGP) and neuraminidase-treated AGP.

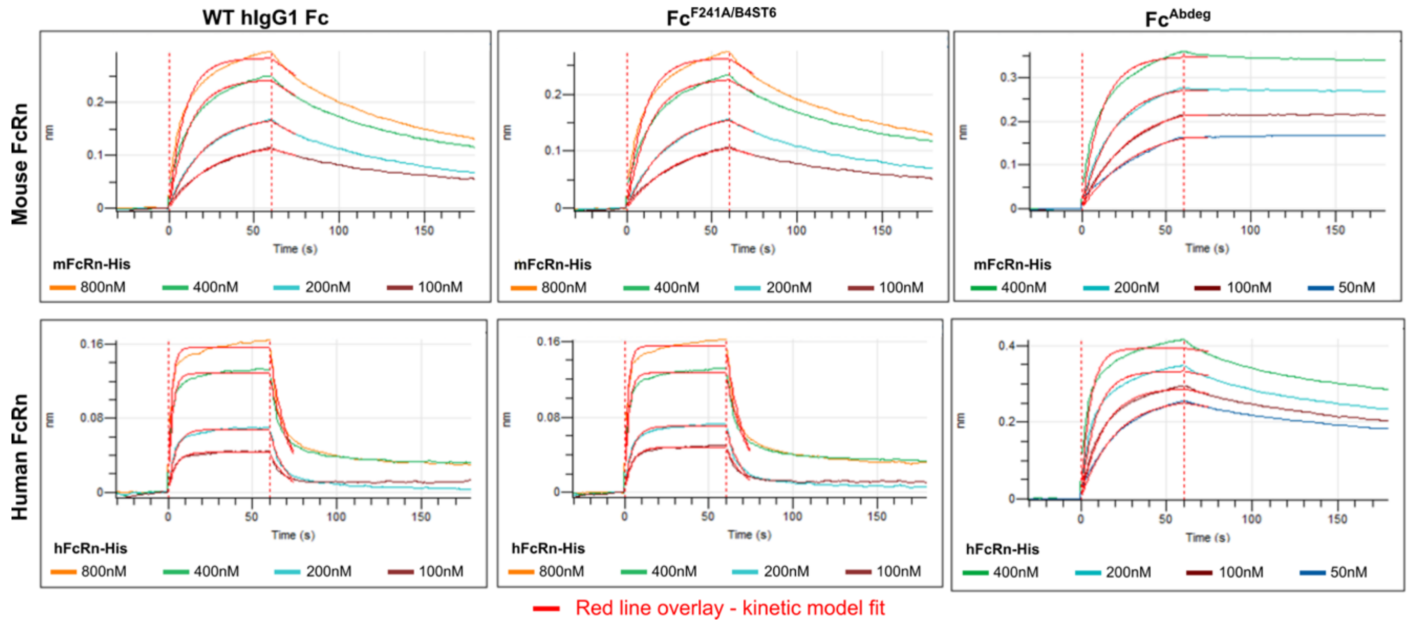

Figure S5. Representative Octet traces (out of 3 replicates each) used to calculate KAs and KDs in main text Figure 3B. Each purified Fc (WT hIgG1 Fc, $Fc^{F241A/B4ST6}$, and Fc^{Abdeg} (Efgartigimod)) was individually captured on anti-human Fc biosensors, then each sensor was dipped in wells containing mouse or human FcRn at a pH of 6.0 for association. Dissociation was performed in a buffer with pH 6.0. From the kinetic model fit curves, KA and KD values were calculated.

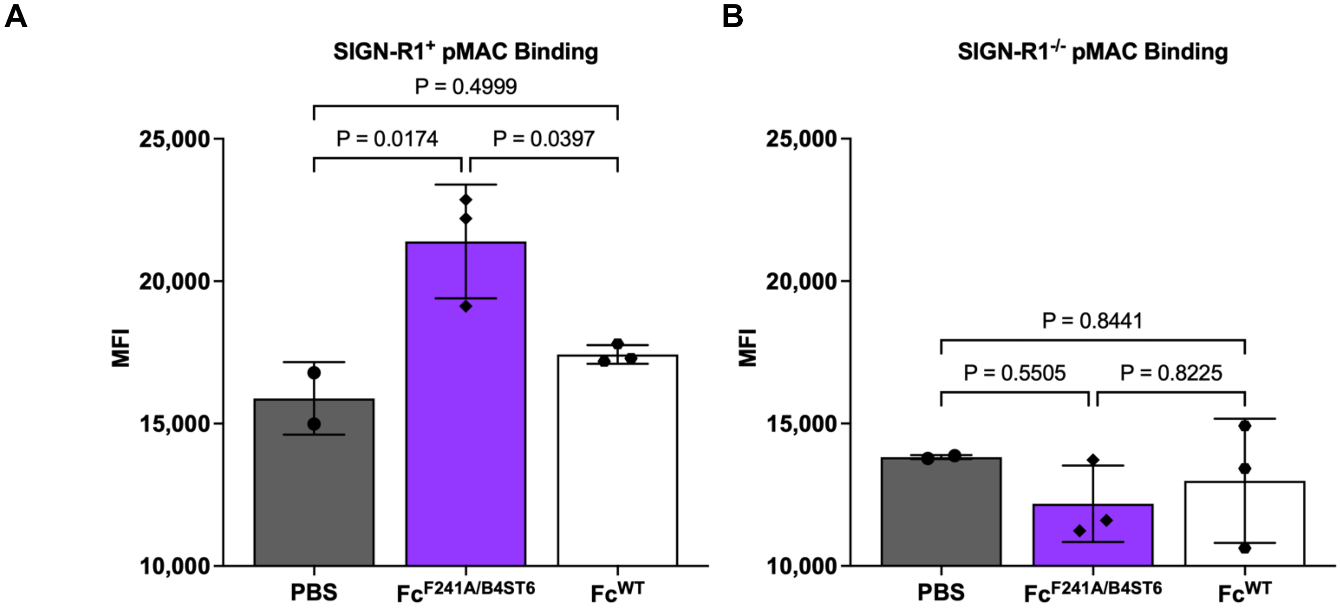

Figure S6. MFI of cell surface binding of PBS, $Fc^{F241A/B4ST6}$, or Fc^{WT} to SIGN-R1^{+} **(A)** and SIGN-R1$^{-/-}$ **(B)** murine peritoneal macrophages (pMACs) as detected by FACS.

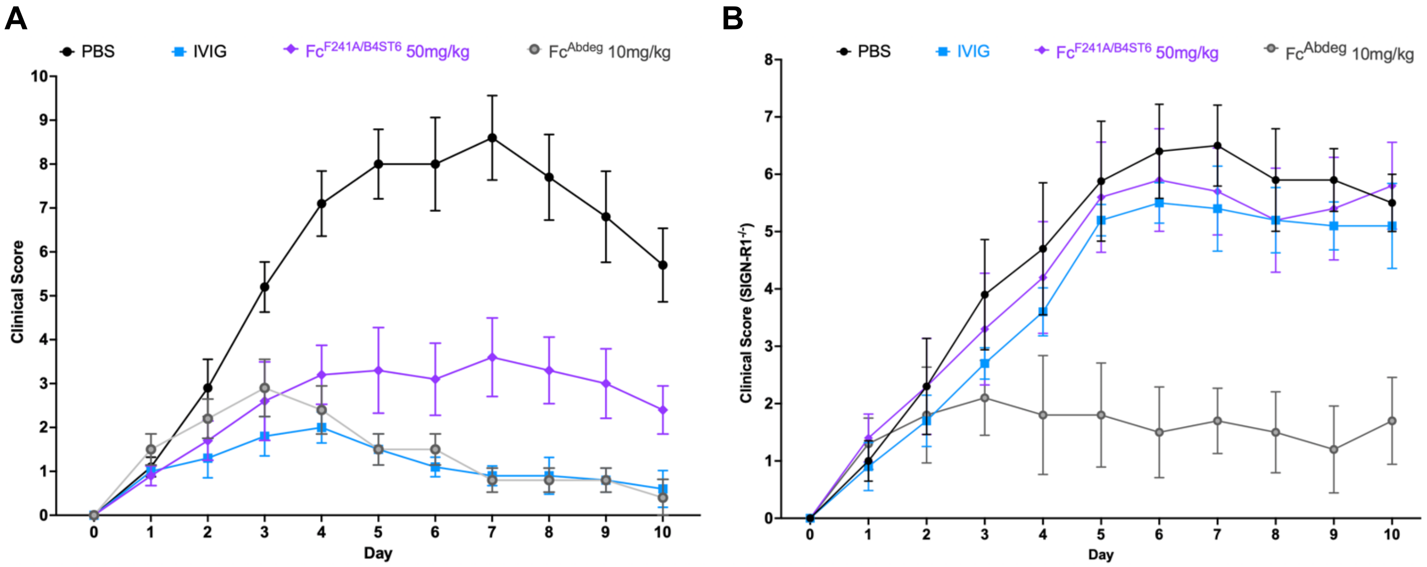

Figure S7. Full time courses of K/BxN experiments shown in main text Figure 3E. (**A**) In female WT C57BL/6 mice (N=5 mice per group), (**B**) in female SIGN-R1-/- mice (N=5 mice per group). Mice were administered PBS (Black circles), IVIG (blue squares), 50mg/kg of $Fc^{F241A/B4ST6}$ (purple diamonds), or 10mg/kg of Fc^{Abdeg} (gray circles) alongside K/BxN serum on day 0. Clinical scores of joint inflammation were recorded for 10 days.

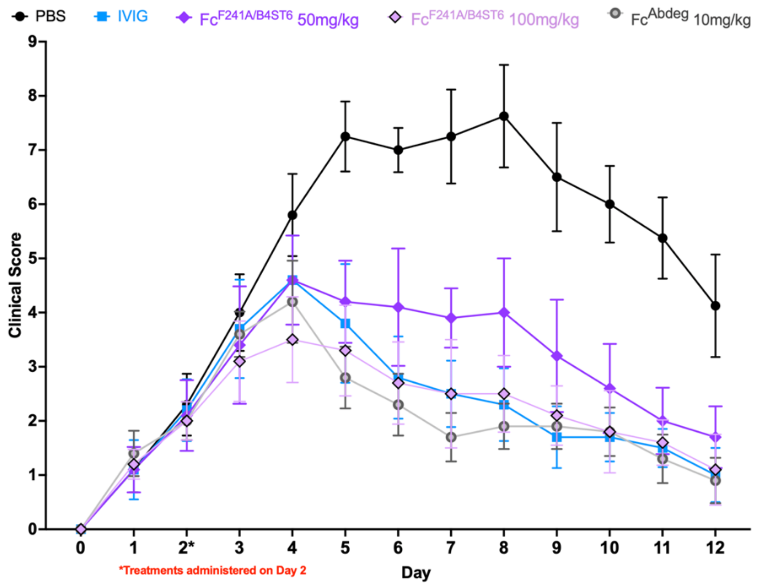

Figure S8. Full time course of K/BxN experiment shown in main text Figure 4A. Female WT C57BL/6 mice (N=4 or 5 mice per group) were administered K/BxN serum on day 0. On day 2, mice were administered PBS (black circles), IVIG (blue squares), 50mg/kg $Fc^{F241A/B4ST6}$ (purple diamonds), 100mg/kg $Fc^{F241A/B4ST6}$ (pink diamonds with outline), or 10mg/kg Fc^{Abdeg} (gray circles). Clinical scores of joint inflammation were recorded for 12 days, starting on day 0.

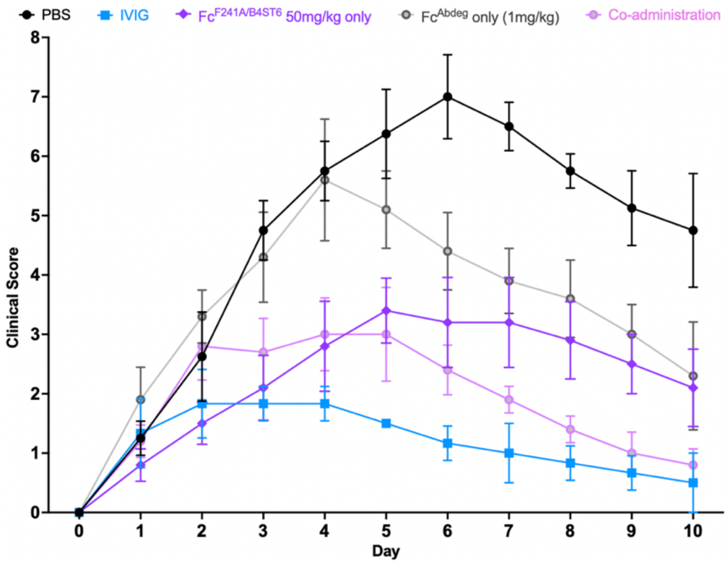

Figure S9. Full time course of K/BxN experiment shown in main text Figure 4D. Female WT C57BL/6 mice (N=3-5 mice per group) were administered PBS (Black circles), IVIG (blue squares), 50mg/kg of $Fc^{F241A/B4ST6}$ (purple diamonds), 1mg/kg of Fc^{Abdeg} (gray circles), or a combination of $Fc^{F241A/B4ST6}$ and Fc^{Abdeg} alongside K/BxN serum on day 0. Clinical scores of joint inflammation were recorded for 10 days.

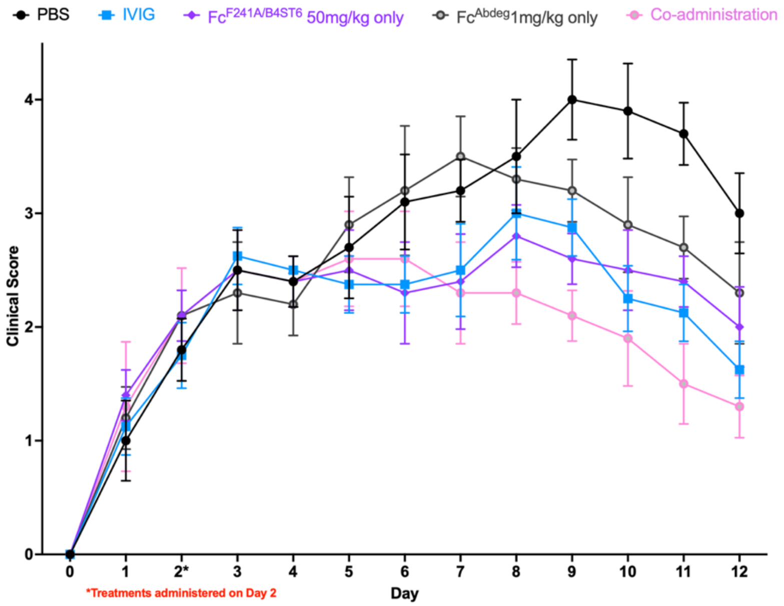

Figure S10. Full time course of K/BxN experiment shown in main text Figure 4E. Female WT C57BL/6 mice (N=5 mice per group) were K/BxN serum on day 0. On day 2, mice were administered PBS (black circles), IVIG (blue squares), 50mg/kg of $Fc^{F241A/B4ST6}$ (purple diamonds), 1mg/kg of Fc^{Abdeg} (gray circles), or a combination of $Fc^{F241A/B4ST6}$ and Fc^{Abdeg} (pink circles) on day 2. Clinical scores of joint inflammation were recorded for 12 days, starting on day 0.
